# Effects of shoulder rotation and table tilt maneuvers on the lumbar and thoracic paramedian acoustic window in older adults: a quasi-experimental study

**DOI:** 10.1016/j.bjane.2026.844769

**Published:** 2026-06-01

**Authors:** Marcos V. Nunes de Souza, Ana M.M. Caetano, Nádia M.C. Duarte, Camila T. Nunes de Souza, Artur J.L. Bezerra, Emmanuelle T.A.G.B.B. Silva

**Affiliations:** a Universidade Federal de Pernambuco, Medical Science Center, Recife, PE, Brazil; b Hospital da Restauração, Department of Anesthesiology, Recife, PE, Brazil

**Keywords:** Aged, Epidural anesthesia, Posterior longitudinal ligament, Spinal anesthesia, Ultrasonography

## Abstract

**Background:**

Shoulder rotation and table tilt have been reported to increase the acoustic window for spinal ultrasonography. However, evidence in older adults remains scarce. This study evaluated their anatomical effects on lumbar (L3–L4) and thoracic (T10–T11) paramedian acoustic windows, quantified by posterior longitudinal ligament (PLL) length, in older adults (≥ 65 years).

**Methods:**

This quasi-experimental study enrolled 45 older adults scheduled for elective surgery at two tertiary hospitals in Brazil. The primary outcome was PLL ultrasonographic length (paramedian sagittal oblique plane) assessed at the L3‒L4 and T10‒T11 interspaces in three positions: F (standard spinal flexion), S (F plus 45° leftward shoulder rotation), and T (F plus 10° dorsal table tilt). Data were analyzed using a Linear Mixed-Effects Model with Bonferroni-adjusted post-hoc contrasts.

**Results:**

The model revealed significant effects of position (p = 0.003) and spinal level (p < 0.001) on PLL length, with no Level × Position interaction (p = 0.693). PLL length was significantly greater at L3–L4 than at T10–T11. Table tilt produced a significant increase relative to the flexion position (difference = 2.2 mm; 95% CI 0.9–3.5; p = 0.002), whereas shoulder rotation did not (difference = 1.2 mm; 95% CI 0.0–2.5; p = 0.158), with homogeneous effects across both levels. Fixed effects explained 27% of total variance (R^2^m = 0.27; R^2^c = 0.55).

**Conclusion:**

Table tilt produced a significant anatomical enlargement of the neuraxial acoustic window at both spinal levels studied, whereas shoulder rotation did not reach statistical significance. These findings establish an anatomical foundation for future trials evaluating their impact on neuraxial procedural outcomes in older adults.

**Trial registration:**

https://ensaiosclinicos.gov.br/rg/RBR-3p6t9pm.

## Introduction

Neuraxial anesthesia offers several key advantages, including maintenance of consciousness, spontaneous ventilation, and airway patency, while providing effective sympathetic and motor blockade, benefits that are particularly relevant for older adults.^1^^,^^2^ However, comorbidities and age-related physiological changes, such as ligament calcification, osteophyte formation, and facet joint hypertrophy, narrow the spinal puncture window, increasing the technical difficulty of neuraxial anesthesia.^3^^,^^4^ Consequently, advanced age directly correlates with a greater number of puncture attempts,^5^ which not only cause patient discomfort but also increase the risk of severe complications, including postdural puncture headache, neuraxial hematoma, and neurological injury.^6^ In this scenario, Ultrasound (US) has become a safe and widely available tool that can help predict and manage difficulties that may arise during neuraxial anesthesia.^7^, ^8^, ^9^, ^10^ Weed et al. identified a correlation between the ease of spinal anesthesia and the size and quality of the Posterior Longitudinal Ligament (PLL), as visualized by US.^11^

Dorsal table tilt and shoulder rotation maneuvers have been reported to increase thoracic PLL length in adults, thereby expanding the spinal acoustic window.^12^, ^13^, ^14^ However, these maneuvers have shown inconsistent results in certain populations, such as pregnant women,^15^ and remain understudied in the elderly.

Additionally, conventional lumbar flexion provides limited expansion of the acoustic window in older patients, especially at L3–L4, where it may not differ from the neutral position.^16^ Given these challenges, maneuvers designed to optimize the acoustic window and facilitate neuraxial blockade in older adults are of utmost clinical relevance. However, before their impact on clinical outcomes can be assessed, their anatomical and biomechanical effects must first be characterized.

The aim of the present study was to evaluate the influence of shoulder rotation and dorsal table tilt on the lumbar (L3‒L4) and thoracic (T10‒T11) acoustic windows in older adults, by measuring PLL length using the Paramedian Sagittal Oblique (PSO) ultrasound plane.

## Methods

### Study design and participants

The study was approved by the Institutional Review Board (IRB) (CAAE: 66608522.1.0000.8807; 66608522.1.3001.5198) and was registered at the Brazilian Registry of Clinical Trials ‒ ReBEC platform (https://ensaiosclinicos.gov.br/rg/RBR-3p6t9pm). This quasi-experimental study was conducted at two tertiary hospitals in Recife, Brazil, and evaluated 50 volunteers (first enrollment: June 19, 2024; last enrollment: December 20, 2024) scheduled for elective surgery.

### Inclusion criteria

The inclusion criteria were ≥ 65 years of age and willingness to provide written informed consent.

### Exclusion criteria

The exclusion criteria were: inability to maintain the designed positions; allergy to ultrasound gel; history of shoulder or spine surgery, trauma, or anomaly (congenital or acquired); and poor ultrasound image quality, defined as a Weed score ≤ 8.^11^ For calculation of the Weed score, each participant was evaluated at four lower lumbar interspaces (L5–S1, L4–L5, L3–L4, L2–L3), bilaterally. Each site was scored 0, 1, or 2 (0 = PLL not visible; 1 = Hazy; 2 = Clear visualization of PLL and its limits), resulting in a total score ranging from 0 to 16 (higher scores, in particular scores above 8, indicate better PLL visualization).^11^

### Data collection and sequence positioning

**Figure 1:**
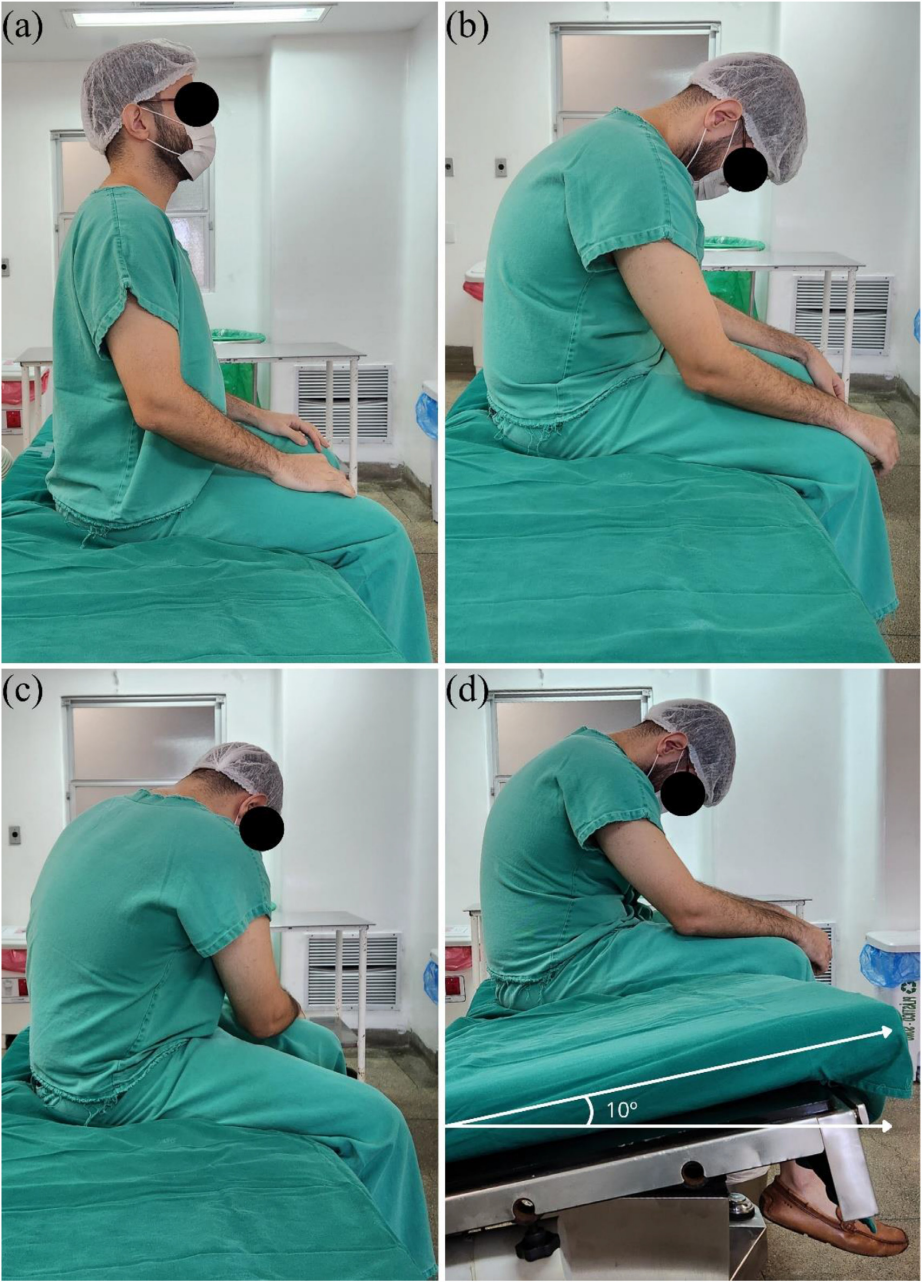
Sitting positions and maneuvers. (a) Normal sitting position. (b) Position F ‒ standard spinal flexed position. Individuals were instructed to relax their shoulders, flex their cervical and lumbar spines, and exaggerate thoracic kyphosis, while resting their arms on their legs. (c) Position S - shoulder rotation maneuver. Same as in position F, and 45° leftward shoulder rotation on the same axis of the vertebral column. (d) Position T – table tilt maneuver. Same as in position F, and 10° dorsal table tilt.

For each study participant, age, sex, weight, height, Body Mass Index (BMI), American Society of Anesthesiologists (ASA) physical status classification, and comorbidities were recorded. Before surgery, each volunteer was seated on the operating table with hips and knees flexed at 90°. Prior to image acquisition, the sonographic quality of the four lower lumbar interspaces was evaluated using the Weed score. This assessment served strictly as an objective exclusion criterion; participants presenting with low-quality image (Weed score ≤ 8) were excluded to ensure the reliability of all subsequent measurements. Spinal ultrasonography in the Paramedian Sagittal Oblique (PSO) plane was then performed at the L3‒L4 and T10‒T11 levels in three consecutive positions (F→S→T), yielding six images per participant.^17^ Each data collection session lasted approximately 15 minutes per participant. This fixed sequence was adopted to ensure accurate assignment of each image to the corresponding position, spinal level, and participant, given that the stored images contained no identifying information. An assistant instructed and assisted patients in maintaining each position, as described below (Fig. 1).

F (Flexion): The shoulders are held in a ‘slouched’ or flexed posture, with cervical and lumbar flexion and exaggeration of the thoracic kyphosis, while the arms rest on the legs.
S (Shoulder rotation): The same as in position F, but with 45° leftward shoulder rotation along the axis of the vertebral column, which was confirmed by a Protractor (Keuwlsoft, London, UK).
T (Table tilt): the patient is placed in position F, but with a 10° dorsal table tilt, confirmed by a Protractor (Keuwlsoft, London, UK).

Shoulder rotation and dorsal table tilt angles were chosen based on previous studies.^12^, ^13^, ^14^, ^15^ We chose the L3‒L4 and T10‒T11 levels because they are commonly used at our institution for administration of anesthesia or analgesia during lower-limb, abdominal, and thoracic surgeries.

### Image acquisition

**Figure 2:**
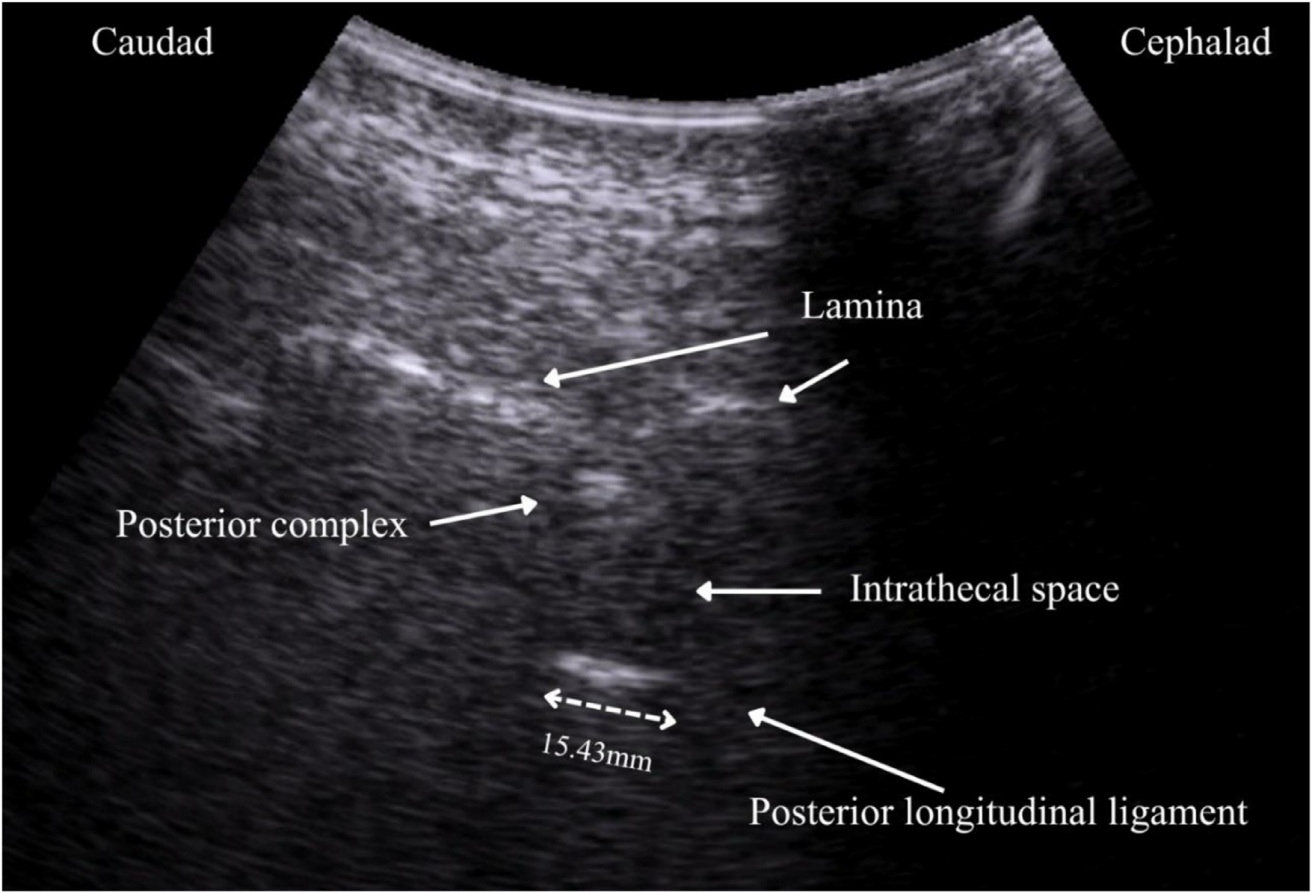
Structures of neuraxial ultrasound on paramedian sagittal oblique plane on L3‒L4 intervertebral space and PLL length along the largest axis. No patient identification, positioning, or level was present in the file name or image.

Starting from the sacral plateau, a scan of the paramedian sagittal oblique plane was performed using a 2–5 MHz curvilinear transducer (GE HealthCare, Chicago, Illinois, USA). Scanning was continued cranially, counting each lamina until the L3‒L4 and T10‒T11 levels were reached. The position was confirmed by manual palpation of spinal processes, beginning with the sacral plateau, and by ultrasonographic identification of the T12 vertebra, which is the first vertebra to articulate with the rib when scanning from bottom to top. The ‘sawtooth’ pattern was used to identify the laminae, while the anterior complex, consisting of the PLL and vertebral body, was visualized as a deeper, brighter structure (Fig. 2).^17^ The best PLL image, optimized by careful probe tilting, was then saved to a USB drive.

### Bias attenuation and image masking

An experienced anesthesiologist (M.S.) with over 100 neuraxial ultrasound scans and three years of practical experience performed all US scans and measurements for the present study. Although the anesthesiologist could not be blinded to the patient’s position, the measurement process was conducted under blinded conditions. An assistant who did not participate in recording the US scans anonymized the stored images (USB drive) by assigning them a random number from 1 to 270 (using a simple random draw) and sent them to the investigator (M.S.) in this new random sequence. The investigator then used these anonymized US images to determine PLL length along the largest axis (Fig. 2). The procedure was executed using the Radiant DICOM Viewer application (Medixant, Poznan, Wielkopolska, Poland). No patient identification, positioning, or level was present in the file name or image (Fig. 2). The examiner compiled all PLL measurements into a spreadsheet, and after completion, the assistant assigned each PLL value to the corresponding participant, spinal level, and position.

### Sample size and statistical analysis

Previous studies enrolled 30‒34 subjects, considering a 1 mm increase in PLL as anatomically meaningful.^12^, ^13^, ^14^, ^15^ Based on data derived from these studies, a statistician calculated that a minimum sample size of 38 (effect size = 0.7, α = 0.05, power = 90%, and 10% dropout) would be ideal for the present study. A target of 50 subjects was therefore established.

A Linear Mixed-Effects Model (LMM) was fitted to account for the hierarchical structure of repeated measurements nested within subjects across positions and spinal levels. Position (F, S, and T; reference: F), spinal level (L3–L4 and T10–T11; reference: L3–L4), and their two-way interaction (Level × Position) were entered as categorical fixed effects, with a random intercept specified for each patient to account for between-subject variability in baseline PLL length. Parameters were estimated using Restricted Maximum Likelihood (REML) with Satterthwaite degrees of freedom approximation. The overall significance of fixed effects was first assessed using Type III F-tests. As the Level × Position interaction was not statistically significant, pairwise position contrasts (S vs. F, T vs. F, and T vs. S) were subsequently computed as marginal comparisons collapsed across spinal levels and adjusted by the Bonferroni method. Effect sizes were reported as marginal R^2^ (R^2^*m*) and conditional R^2^ (R^2^*c*), reflecting variance explained by fixed effects alone and by the full model, respectively.

Model assumptions were satisfied. Residual normality was confirmed by the Shapiro-Wilk test (p = 0.403) and quantile-quantile plots, with no evidence of heteroscedasticity on inspection of residuals versus fitted values. The random effects followed an approximately normal distribution.

The primary outcome was PLL length at the L3–L4 and T10–T11 interspaces across positions F, S, and T. No secondary outcomes were pre-specified. All analyses were performed in R software (version 4.4.2, R Core Team, Vienna, Austria).

## Results

**Figure 3:**
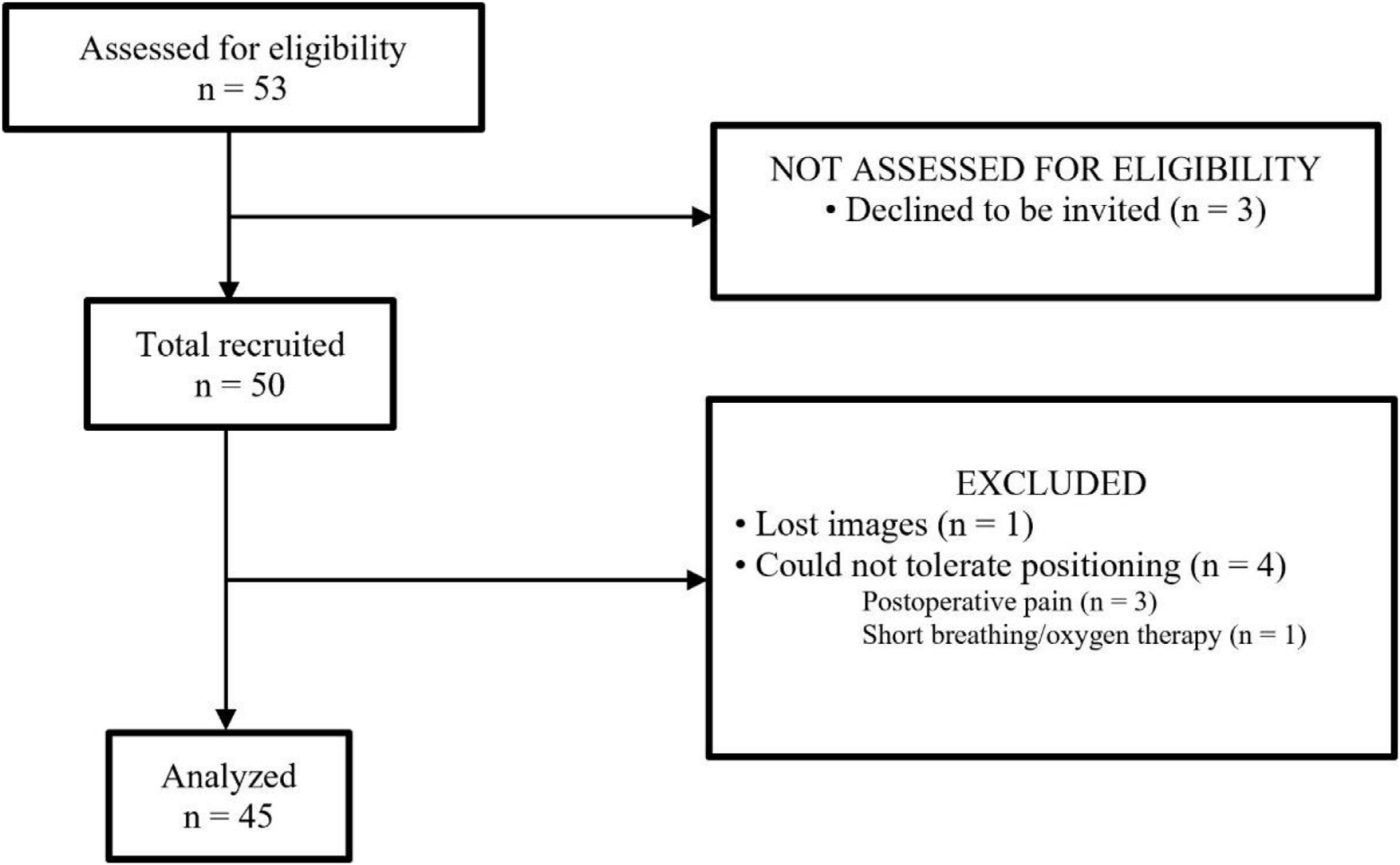
Enrollment of patients in the study.

Figure 3 summarizes the enrollment process. Initially, 50 volunteers met the study eligibility criteria. However, four had to be excluded from the present study because they could not maintain the required positions: of these, three reported pain related to a recent unrelated surgery, and one required supplemental oxygen and experienced shortness of breath. In addition, imaging data for one participant were lost due to a power outage. Baseline characteristics of the five excluded participants were not formally compared with those of the included sample owing to the small number of exclusions; however, no systematic differences in age, sex, or ASA classification were apparent from available records. Thus, a total of 45 participants were included in the final analysis, and their demographic characteristics are presented in Table 1.

**Table 1:** Demographic characteristics of participants*.*

Characteristic	Data (n = 45)
Sex, n (%)	
Male	22 (49)
Female	23 (51)
Age, years (mean ± SD)	72.0 ± 6.0
Height, m (mean ± SD)	1.63 ± 0.09
Body mass index, kg.m^-2^ (mean ± SD)	24.7 ± 5.4
Weight, kg (mean ± SD)	65.4 ± 13.3
ASA classification^a^, n (%)	
I	2 (4)
II	14 (31)
III	28 (62)
IV	1 (2)
Comorbidities^b^, n (%)	
Diabetes mellitus	32 (71)
Systemic arterial hypertension	32 (71)
Peripheral arterial disease	19 (42)
Smoking	19 (42)
Overweight	14 (31)
Alcoholism	9 (20)
Obesity	6 (13)
Coronary artery disease	3 (7)
Cancer	2 (4)
Others	9 (20)

ASA, American Society of Anesthesiologists. Data are expressed as mean ± SD, and number (%).

a Sum of percentages was 99% due to rounding.

b Sum of percentages exceeded 100% because it is a prevalence measure, and each participant might have more than one comorbidity.

Four measurements (two at L3–L4 and two at T10–T11) yielded a PLL length of 0 mm in position F, as the PLL could not be visualized despite repeated scanning; notably, in these same participants, the PLL became visible after application of the maneuvers.

**Figure 4:**
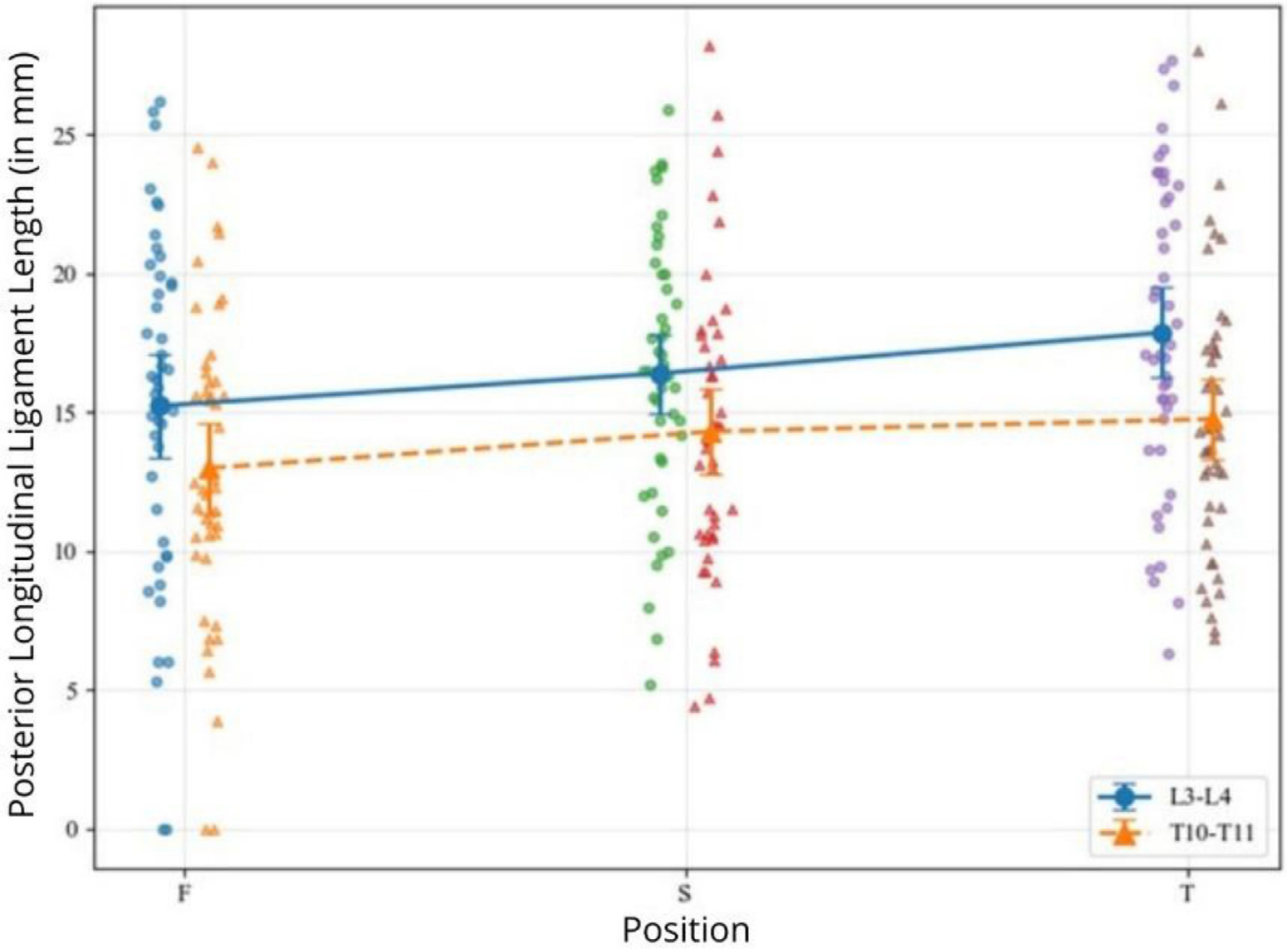
Estimated Marginal Means and 95% confidence interval of PLL length over position F (standard spinal Flexed position), S (Shoulder rotation maneuver), and T (Table tilt maneuver) for each level.

Table 2 and Figure 4 summarize the key outcomes. The LMM revealed significant overall effects of position and spinal level on PLL length, with no significant Level × Position interaction (p = 0.693), indicating that the effects of the maneuvers were statistically homogeneous across spinal levels. In the post-hoc analysis, PLL length was significantly greater at L3–L4 than at T10–T11. Table tilt was the only maneuver to produce a significant increase in PLL length relative to the flexion position, an effect considered applicable to both spinal levels; shoulder rotation did not reach statistical significance. The difference between table tilt and shoulder rotation was also not statistically significant.

**Table 2:** Linear mixed-effects model results: estimated marginal means of PLL length, omnibus F-tests, and post-hoc pairwise comparisons (n = 45).

Estimated Marginal Means				
Level	Position			
	F [95% CI]	S [95% CI]		T [95% CI]
L3-L4 (mm)	15.2 ± 0.8 [13.6, 16.9]	16.4 ± 0.8 [14.8, 18.0]		17.9 ± 0.8 [16.2, 19.5]
T10-T11 (mm)	13.0 ± 0.8 [11.3, 14.6]	14.3 ± 0.8 [12.7, 15.9]		14.8 ± 0.8 [13.1, 16.4]

Fixed Effects Omnibus Tests				
	F-statistic	df	df (res)	p-value
**Position**	5.976	2	220	**0.003**
**Level**	22.536	1	220	**< 0.001**
**Position × Level**	0.367	2	220	0.693

Post-hoc pairwise comparison^a^				
Level	Difference [95% CI]			p-value Bonferroni
L3-L4 – T10-T11 (mm)	2.5 ± 0.5 [1.45, 3.51]			**< 0.001**
**Position**				
S – F (mm)	1.2 ± 0.6 [0.0, 2.5]			0.158
T – F (mm)	2.2 ± 0.6 [0.9, 3.5]			**0.002**
T – S (mm)	1.0 ± 0.6 [-0.3, 2.2]			0.405

PLL, Posterior Longitudinal Ligament; 95% CI, 95% Confidence Interval; df, Degrees of freedom; df(res), Residual degrees of freedom; F, Standard spinal flexion; S, 45° leftward shoulder rotation; T, 10° dorsal table tilt.

Coefficients of determination R^2^m = 0.27; R^2^c = 0.55.

Data are expressed as mean ± SE; 95% Confidence Intervals are model-derived from the Linear Mixed-Effects Model.

a Post-hoc comparisons for Position are reported as marginal means collapsed across spinal levels, as the Level × Position interaction was not statistically significant (p = 0.693); p-values adjusted by Bonferroni correction.

Fixed effects explained 27% of the total variance in PLL length (R^2^m = 0.27), rising to 55% when between-subject variability was accounted for (R^2^c = 0.55). The random intercept variance was 11.2 mm^2^ (SD = 3.34 mm), reflecting substantial individual variability in baseline PLL length across patients.

## Discussion

In the present study, we hypothesized that shoulder rotation and table tilt maneuvers would enlarge the acoustic window at the lumbar (L3‒L4) and thoracic (T10‒T11) levels in older adults. The LMM revealed a significant overall effect of position on PLL length, driven by the table tilt maneuver, which reached statistical significance in the post-hoc analysis. The Level × Position interaction was not significant, indicating that the anatomical response to the maneuvers was statistically homogeneous across spinal levels; the effect of table tilt was therefore interpreted as applicable to both L3–L4 and T10–T11. Shoulder rotation did not reach statistical significance after Bonferroni correction. These findings establish an anatomical foundation for future clinical trials evaluating the impact of these maneuvers on clinical outcomes in older adults.

Sebbag et al. evaluated shoulder rotation and table tilt in full-term pregnant women at the L3–L4 interspace, but found no significant increase in PLL dimensions, likely owing to the limited lumbar flexion imposed by the gravid uterus.^15^ In contrast, the present study demonstrated a statistically significant anatomical effect of table tilt across both spinal levels, highlighting the need for further research in challenging patient populations.

Unlike previous investigations, which reported significant increases in PLL length following shoulder rotation at thoracic levels,^12^, ^13^, ^14^ the present study found no statistically significant effect of this maneuver at either spinal level. In contrast, table tilt resulted in an increase in PLL length consistent with or exceeding values reported in prior investigations,^12^, ^13^, ^14^ suggesting that this maneuver may be particularly appropriate for elderly patients.

The significantly greater PLL length at L3–L4 compared with T10–T11 (p < 0.001) is consistent with the known anatomical differences between lumbar and thoracic vertebral morphology, with wider intervertebral spaces at lower spinal levels.

Biomechanically, shoulder rotation induces vertebral torsion by rotating the upper trunk with respect to the hips, which are held fixed, resulting in a more pronounced enlargement of the interlaminar spaces at higher thoracic levels.^18^^,^^19^ Therefore, the diminished and non-significant response at both levels may also be attributed to reduced mobility, dehydration, and age-related narrowing of the intervertebral spaces.^3^^,^^4^ Table tilt, by contrast, enhances both lumbar and thoracic flexion simultaneously, promoting a more uniform expansion of the intervertebral spaces across levels.^18^^,^^19^

A difference of at least 1 mm in PLL length has been proposed as an anatomically meaningful threshold, approximating the outer diameter of a standard epidural needle.^12^, ^13^, ^14^ In the present study, all observed mean differences between positions exceeded this threshold at both spinal levels; however, only table tilt reached statistical significance. Future studies with larger sample sizes may help clarify whether shoulder rotation produces a consistently detectable anatomical effect in this population.

The effects of the maneuvers observed in the present study can be further characterized by the effect size metrics derived from the LMM. Fixed effects (spinal level and position) explained 27% of the total variance in PLL length (R^2^m = 0.27), a moderate proportion indicating that position and vertebral level are meaningful determinants of PLL length. When individual between-subject variability was incorporated through the random intercept, the model explained 55% of total variance (R^2^c = 0.55). The gap between R^2^m and R^2^c reflects the substantial contribution of individual anatomical differences, quantified as a random intercept variance of 11.2 mm^2^ (SD = 3.34 mm), underscoring that baseline PLL dimensions vary considerably across older adults, independently of position or spinal level.

Interestingly, while the PLL could not be identified in four initial images obtained in position F, it became visible after applying the proposed maneuvers. This finding highlights the potential of these techniques; however, whether this translates into any procedural advantage remains to be established in future clinical studies.

The present study has some limitations. All ultrasound examinations were performed by a single experienced investigator (over 100 US scans), precluding interobserver reliability analysis. This limitation reduces the external validity of the present findings, given the operator-dependent nature of ultrasound imaging. The same investigator who performed the ultrasound scans subsequently measured the PLL lengths; as blinding of the examiner was not feasible, potential measurement bias was minimized by anonymizing the images and presenting them in a random sequence, ensuring the examiner was unaware of patient identity, maneuver, or spinal level. The fixed sequential order of the positions (F→S→T) without randomization may have introduced an order bias in both directions: while progressive muscle relaxation over time could have favored a greater expansion of the acoustic window during the final maneuvers, cumulative fatigue arising from sustained non-physiological positions could have conversely hindered the volunteers' ability to maintain the optimal posture.

Although fatigue or discomfort could interfere with maintaining the required positions, 90% of the study participants tolerated the maneuvers well. Among the 50 volunteers who met the inclusion criteria, five were excluded: four due to pain or discomfort that prevented proper positioning, and one because of image loss during a power outage. Based on the present study, the maneuvers appear to be well-tolerated by older adults, although specific clinical conditions may limit their general applicability. Nevertheless, the final sample size still exceeded the minimum required for statistical validity.

Finally, and most importantly, the present study assessed PLL length as a surrogate anatomical marker and was not designed to evaluate clinical outcomes. Whether enlargement of the acoustic window translates into improved procedural outcomes requires prospective clinical investigation.

The present study is notable for evaluating an elderly patient population, which has often been excluded from previous investigations.^12^, ^13^, ^14^, ^15^ Older individuals have biomechanical constraints that may limit expansion of the intervertebral spaces and reduce the effectiveness of the maneuvers. Nevertheless, table tilt produced a statistically significant and anatomically meaningful increase in PLL length applicable to both spinal levels studied.

The study was conducted at two tertiary public hospitals within a developing country, reflecting a population with limited access to health-promoting resources and treatments. This is supported by the high prevalence of patients classified as ASA III-IV, indicating significant comorbidity burden, which may have adversely affected spinal mobility and attenuated the observed effects. Accordingly, the present findings may represent a conservative estimate of the anatomical response to table tilt, and this response may be more pronounced in healthier elderly individuals; however, whether comorbidity burden directly affects PLL dimensions remains to be investigated.

## Conclusion

Table tilt enlarged the acoustic window, as reflected by PLL enlargement, at both the lumbar (L3–L4) and thoracic (T10–T11) levels in older adults. Shoulder rotation did not significantly increase PLL length at either level. As the present study assessed PLL length as a surrogate anatomical marker, these findings provide anatomical evidence that table tilt may enlarge the neuraxial acoustic window in older adults. Future clinical trials should assess the ability of these maneuvers to reduce the technical challenges associated with the administration of neuraxial anesthesia and related complication rates.

## Declaration of generative AI and AI-assisted technologies in the manuscript preparation process

During the preparation of this work the author(s) used Chat GPT and Claude in order to improve fluency, language and readability. After using this tool/service, the author(s) reviewed and edited the content as needed and take(s) full responsibility for the content of the published article.

## Data availability statement

The datasets generated and/or analyzed during the current study are available from the corresponding author upon reasonable request.

## Funding

This research did not receive any specific funding from funding agencies in the public, private, or non-profit sectors.

## Acknowledgements

We are thankful to Kamila Cunha for providing assistance during data collection.

## CRediT authorship contribution statement

**Marcos V. Nunes de Souza:** Conceptualization, Data curation, Formal analysis, Funding acquisition, Investigation, Methodology, Project administration, Resources, Software, Supervision, Validation, Visualization, Writing – original draft, Writing – review & editing. **Ana M.M. Caetano:** Conceptualization, Methodology, Supervision, Validation, Writing – review & editing. **Nádia M.C. Duarte:** Conceptualization, Methodology, Writing – review & editing. **Camila T. Nunes de Souza:** Data curation, Formal analysis, Investigation, Resources, Visualization, Writing – original draft, Writing – review & editing. **Artur J.L. Bezerra:** Data curation, Investigation, Writing – review & editing. **Emmanuelle T.A.G.B.B. Silva:** Conceptualization, Methodology, Project administration, Supervision, Validation, Writing – review & editing.

## Conflicts of interest

The authors declare no conflicts of interest.
